# Solid Indeterminate Pulmonary Nodules Less Than or Equal to 250 mm^3^: Application of the Updated Fleischner Society Guidelines in Clinical Practice

**DOI:** 10.1155/2019/7218258

**Published:** 2019-01-03

**Authors:** Andrea Borghesi, Silvia Michelini, Giorgio Nocivelli, Mario Silva, Alessandra Scrimieri, Stefania Pezzotti, Roberto Maroldi, Davide Farina

**Affiliations:** ^1^Department of Radiology, University and Spedali Civili of Brescia, Piazzale Spedali Civili 1, 25123 Brescia, Italy; ^2^Department of Radiology, Fondazione Poliambulanza Istituto Ospedaliero, Via Leonida Bissolati, 57, 25124 Brescia, Italy; ^3^Section of Radiology, Department of Medicine and Surgery, University of Parma, Pad. Barbieri, Via Gramsci 14, 43126 Parma, Italy

## Abstract

**Background:**

The latest version of the Fleischner Society guidelines for management of incidental pulmonary nodules was published in 2017. The main purpose of these guidelines is to reduce the number of unnecessary computed tomography (CT) examinations during the follow-up of small indeterminate nodules.

**Objective:**

The present study aimed to evaluate the performance of these guidelines for management of solid indeterminate pulmonary nodules (SIPNs) ≤ 250 mm^3^.

**Materials and Methods:**

During a 7-year period, we retrospectively reviewed the chest CT scans of 672 consecutive patients with SIPNs. The study sample was selected according to the following inclusion criteria: solitary SIPN; diameter ≥ 3 mm; volume ≤ 250 mm^3^; two or more CT scans performed with the same scanner and same acquisition/reconstruction protocol; thin-section 1-mm images in DICOM format; histologic diagnosis or follow-up ≥ 2 years; and no oncological history. Applying these criteria, a total of 27 patients with single SIPNs ≤ 250 mm^3^ were enrolled. For each SIPN, the volume and doubling time were calculated using semiautomatic software throughout the follow-up period. For each SIPN, we applied the Fleischner Society guidelines, and the recommended management was compared to what was actually done.

**Results:**

A significant volumetric increase was detected in 5/27 (18.5%) SIPNs; all growing nodules were observed in high-risk patients. In these SIPNs, a histologic diagnosis of malignancy was obtained. Applying the Fleischner Society recommendations, all five malignant nodules would have been identified. None of the SIPNs < 100 mm^3^ in low-risk patients showed significant growth during the follow-up period. The application of the new guidelines would have led to a significant reduction in follow-up CT examinations (Hodges-Lehmann median difference, -2 CT scans; p = 0.0001).

**Conclusion:**

The application of the updated Fleischner Society guidelines has been shown to be effective in the management of SIPNs ≤ 250 mm^3^ with a significant reduction in radiation dose.

## 1. Introduction

A pulmonary nodule is defined as a focal opacity measuring less than 3 cm in diameter [1]. Pulmonary nodules are a frequent incidental finding on routine chest computed tomography (CT) [1, 2], but their prevalence in the general population is not known precisely. Pulmonary nodules smaller than 8 mm in diameter are considered small nodules [3], while nodules ranging between 3 and 5 mm in diameter are classified as very small nodules. These very small nodules, so called “ditzels” (i.e.,* little things that could mean a lot*) [4, 5], represent approximately 50% of all pulmonary nodules detected on CT [6]. Micronodule is a term that should be reserved only for nodules smaller than 3 mm [7]. In patients without malignancy, these micronodules should be considered benign until proven otherwise.

The improvement in spatial resolution and broad availability of multidetector-row CT (MDCT) has significantly increased the detection rate of pulmonary nodules, particularly those smaller than 8 mm. Many small pulmonary nodules are benign (i.e., hamartomas or granulomas) [1, 2]. However, the majority of these nodules remain indeterminate. In fact, a confident diagnosis of benignity can be formulated only for fully calcified and fat-containing nodules.

The malignancy rate of small pulmonary nodules is very low but not zero. In their high-risk cohort, Swensen et al. [8] identified a malignancy rate of 1% among 3072 nodules smaller than 8 mm. Therefore, the most challenging category of nodules is represented by indeterminate pulmonary nodules (i.e., noncalcified and non-fat-containing) with a diameter ranging from 3 to 8 mm.

The goal of the radiological evaluation of small pulmonary nodules is to differentiate benign from malignant lesions as noninvasively and as accurately as possible. In clinical practice, small indeterminate nodules usually require monitoring with serial chest CT scans for a minimum of 2 years [9]. For these nodules, fluorine-18 FDG PET is inaccurate for discriminating between benign and malignant lesions. Therefore, the only indicator of malignancy is the growth rate.

The first version of the Fleischner Society guidelines for management of small pulmonary nodules was published in 2005. In this statement, only solitary solid nodules were considered, and the recommendations were based on nodule diameter and patient risk factors for lung cancer [9]. In the latest version of the Fleischner Society guidelines for management of incidental pulmonary nodules, which was published at the beginning of 2017 [10], the recommendations include both solid and subsolid nodules and, in addition to nodule diameter, also consider other nodule features, such as volume, multiplicity, morphology, and location.

The main purpose of these updated guidelines is to reduce the number of unnecessary CT examinations during the follow-up of small indeterminate nodules while providing radiologists and clinicians with greater power in the decision-making process.

The main purpose of the present study was to evaluate the performance of the updated Fleischner Society guidelines in a group of solitary solid indeterminate pulmonary nodules (SIPNs) ≤ 250 mm^3^ that were incidentally detected on routine chest CT scans.

## 2. Materials and Methods

### 2.1. Patient and Nodule Selection

During a 7-year period, from January 2005 to January 2012, a retrospective search was performed in the department radiology information system/picture archiving and communication system (RIS/PACS) to retrieve all MDCT reports containing findings indicative of SIPNs ≤ 8 mm in diameter.

The search identified a total of 672 patients with one or more SIPNs ≤ 8 mm in diameter. The images contained in these MDCT reports were reviewed by an experienced radiologist (A.B. who had 14 years of experience in thoracic imaging), and the study sample was selected according to the following inclusion criteria: (a) solitary SIPN (i.e., noncalcified and non-fat-containing); (b) nodule diameter (i.e., the average between the longest axis and its perpendicular short axis on the image with the largest cross-sectional area of the lesion) ≥ 3 mm; (c) nodule volume ≤ 250 mm^3^; (d) two or more MDCT examinations performed with the same scanner and the same acquisition/reconstruction protocol; (e) thin-section 1-mm lung window images in DICOM format; (f) histologic diagnosis or follow-up more than two years. Patients who were younger than 35 years, those who were immunocompromised, and those who had an oncologic history or previous granulomatous disease were excluded.

Applying these criteria, a total of 27 patients with single SIPNs ≤ 250 mm^3^ were enrolled in the study. The selected patients were classified as having a low or high risk of lung cancer based on their smoking history and exposure to other carcinogens. Patients with a minimal or absent history of smoking and no occupational exposure to carcinogens (such as asbestos, silica, coal mine dust, beryllium, hard-metal dust, or uranium) were classified as low risk for lung cancer. On the other hand, patients with a smoking history and/or occupational exposure to carcinogens were classified as high risk for lung cancer. The characteristics of the study patients are listed in Table 1.

### 2.2. Image Acquisition

All chest CT examinations were performed with the same MDCT scanner (Somatom Sensation 16; Siemens, Germany) and the same acquisition/reconstruction protocol. The MDCT scanner used the following parameters: collimation, 16 × 0.75 mm; beam pitch, 1.0; rotation time, 0.5 s; tube voltage, 120 kVp; and tube current, 180 mAs. The acquisition, extending from the lung apex to the lung base, was performed at end inspiration. The volume was reconstructed as 1-mm thick sections, applying a sharp reconstruction algorithm and preset windowing suited for lung parenchyma assessment. No contrast material was used.

### 2.3. Image Analysis

For each SIPN, the axial diameter (i.e., the long and short axes on the image with the largest cross-sectional area) and the volume at baseline and at the last follow-up CT scan were calculated using three-dimensional semiautomatic software (SAT module, classic version, Terarecon Inc., United States). The volumetric variation in each SIPN between baseline and the last CT scan was automatically calculated by matching the volumes. The volume doubling time (VDT) was generated only when a positive variation was detected by the software. A volumetric increase of more than 25% [11] with a VDT < 600 days was considered clinically significant.

Prior to the computerized analysis, visual assessment of the nodule margins and lobe location on the baseline CT scan was performed.

For each SIPN, we applied the updated Fleischner Society guidelines (*in their less conservative version*) (Table 2), and the recommended management was compared to what was actually done.

The computerized analysis and visual assessment of the nodule margins and lobe location were performed by the same experienced thoracic radiologist (A.B.).

The present study was retrospective, and patient management was not altered; thus, no specific consent was required. However, informed consent for the use of personal data was obtained from all patients.

### 2.4. Statistical Analysis

The data are presented as numbers (%) or the mean ± standard deviation for normally distributed data or as the median and interquartile range (IQR) for not normally distributed data. A chi-square test was used to analyze differences in the distribution of the nodule characteristics (size, margins, and location) between the low- and high-risk groups. A chi-square test was also used to investigate significant relationships between nodule outcome and certain independent variables (such as sex, patient risk status, nodule size, nodule margins, and nodule location). The Wilcoxon matched-pairs test was used to analyze the difference between the number of CT scans actually performed and the number of CT scans required according to the new Fleischner Society guidelines.

Statistical analysis was performed using dedicated software (MedCalc Software Version 18.2.1). p values <0.05 were considered statistically significant.

## 3. Results

Segmentation and computerized analysis were successfully performed in all 27 SIPNs.

At the baseline MDCT, 16/27 (59.3%) SIPNs had a volume < 100 mm^3^ (8 SIPNs in low-risk patients and 8 SIPNs in high-risk patients), and 11/27 (40.7%) SIPNs had a volume ranging between 100 and 250 mm^3^ (8 SIPNs in low-risk patients and 3 SIPNs in high-risk patients). No statistically significant differences were observed in the distribution of the nodule characteristics (size, margins, and location) between the low- and high-risk groups (Table 3).

A significant increase in volume (more than 25% with a VDT < 600 days) between baseline and the last CT scan was observed in 5/27 (18.5%) SIPNs (2 SIPNs < 100 mm^3^ and 3 SIPNs measuring between 100 and 250 mm^3^). In this group exhibiting growth, the volume increased from 71% to 292% (mean, 173 ± 101%) with VDT ranging from 93 to 447 days (mean, 257 ± 164 days); the time interval between baseline and the last CT scan ranged from 165 to 411 days (mean, 287 ± 108 days). These growing nodules were observed in high-risk patients and were surgically removed with a histological diagnosis of non-small cell lung cancer. The characteristics and histological features of the malignant nodules are listed in Table 4.

**Figure 1:**
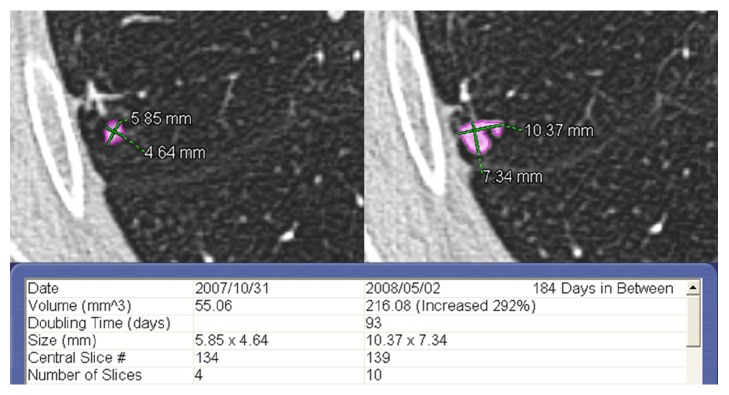
Solid pulmonary nodule smaller than 100 mm^3^ with smooth margins located in the right upper lobe in a 71-year-old high-risk male patient. Baseline (left) and follow-up CT scans (right). The interval between the two CT examinations was 184 days. The software calculated significant growth between the baseline and follow-up CT scans with a relative volume variation of 292% (from 55 mm^3^ to 216 mm^3^) and a volume doubling time of 93 days. The nodule was surgically removed and proven to be a pulmonary adenocarcinoma. In this case, the new Fleischner Society guidelines would have recommended a follow-up CT examination at 12 months.

Applying the new Fleischner Society guidelines, all 5 malignant nodules would have been identified: in 2/5 (40%) nodules, the radiological diagnosis would have been formulated with similar timing (within 45 days); in 2/5 (40%) nodules, there would have been an earlier diagnosis (136 and 180 days, respectively); and in the remaining nodule (20%), there would have been a diagnostic delay of 181 days (see Figure 1).

**Figure 2:**
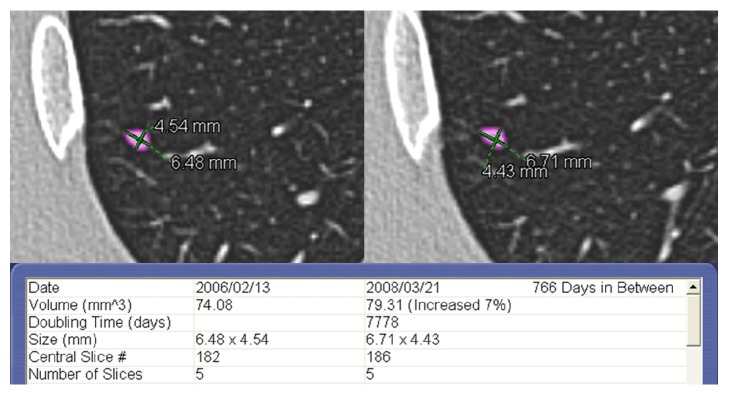
Solid pulmonary nodule smaller than 100 mm^3^ with smooth margins located in the right lower lobe in a 36-year-old low-risk male patient. Baseline (left) and last follow-up CT scans (right). The interval between the two CT examinations was 766 days. The software did not show significant growth of the nodule (relative volume variation of 7% with a volume doubling time of 21 years). The unequivocal nodule stability after more than 2 years indicates the benign nature of the lesion. In this case, the new Fleischner Society guidelines would not have recommended a follow-up CT scan.

None of the SIPNs < 100 mm^3^ identified in low-risk patients showed significant growth during the follow-up period (see Figure 2).

The application of the new Fleischner Society guidelines in our sample would have led to a significant reduction in the number of follow-up CT examinations (Hodges-Lehmann median difference, -2 CT scans; 95% confidence interval, -2.5 to -1.5; p = 0.0001).

A statistically significant relationship was observed between nodule outcome, patient risk status, and nodule margins (Table 5). On the other hand, no statistically significant relationships were observed among nodule outcome, sex, nodule size, and nodule location (Table 5). However, considering only the high-risk group, we found a significant relationship between nodule outcome and nodule size (p = 0.034), as all three nodules with volumes ranging from 100 mm^3^ to 250 mm^3^ were malignant (Table 4). Moreover, we observed that 4/5 (80%) malignant nodules were located in the upper lobe (Table 4).

## 4. Discussion

Pulmonary nodules are classified as solid or subsolid nodules based on their consistency on thin-section CT. Subsolid nodules are further classified as part-solid or pure ground-glass nodules according to the presence or absence of a solid component within the lesion [12, 13].

Among all pulmonary nodules incidentally detected on MDCT, solid nodules are the most frequent, followed by pure ground-glass nodules and part-solid nodules [5, 14]. The malignancy rate of solid nodules is very low, particularly in nodules smaller than 8 mm [8]. Therefore, the characterization of these small solid nodules remains a diagnostic challenge for radiologists.

The size and morphology (especially nodule margins) are considered the main parameters to estimate lung cancer risk [5, 15]. However, small solid nodules frequently remain indeterminate, and a follow-up CT examination is usually recommended to exclude growth.

In the NELSON trial, the 2-year risk of lung cancer in nodules smaller than 100 mm^3^ was 0.6% and did not differ significantly from the 2-year lung cancer risk for participants without baseline lung nodules [16, 17]. On the basis of this low risk of malignancy, the updated Fleischner Society guidelines suggest no routine CT follow-up for solid nodules with a volume < 100 mm^3^ (or smaller than 6 mm) [10, 18]. This recommendation has been proposed for all patients, regardless of their clinical risk status. The threshold size of 100 mm^3^ was established to exclude from CT follow-up all solid nodules with a cancer risk less than 1% [10, 18]. However, the presence of a suspicious morphology (such as spiculation), an upper lobe location or both in nodules < 100 mm^3^, increases the cancer risk up to 5%. Thus, high-risk patients with solid nodules < 100 mm^3^ with a suspicious morphology and/or an upper lobe location should undergo a CT scan after one year [10, 18].

With regard to solitary SIPNs ranging from 100 mm^3^ to 250 mm^3^, the new guidelines recommend an initial CT follow-up at 6-12 months [10, 18]. For patients at low risk, a single follow-up is usually sufficient to demonstrate the stability of the nodule [10, 18]. However, when a suspicious morphology is present or volumetric stability is uncertain, an additional CT follow-up at 18-24 months should be considered [10, 18]. Conversely, for patients at high risk, a second follow-up at 18-24 months is strongly recommended because, in this size range, the cancer risk grows up to 2% [10, 15–19].

To the best of our knowledge, the present study is the first to evaluate the performance of the updated Fleischner Society guidelines in a group of solitary SIPNs ≤ 250 mm^3^ incidentally detected in routine clinical settings. Our study found that the retrospective application of the new Fleischner Society guidelines has proven to be effective in the management of SIPNs ≤ 250 mm^3^, as all malignant nodules would have been identified, and none of the SIPNs < 100 mm3 in low-risk patients showed significant growth during the follow-up period. Moreover, we observed that the new guidelines would have led to a significant reduction in the number of CT examinations performed, resulting in a significant reduction in the radiation dose delivered to the patients.

The only critical aspect in the application of the new guidelines was observed in a malignant nodule with a baseline volume of 55 mm^3^ that would have been identified with a delay of 6 months (see case 2 in Table 4 and Figure 1). However, considering the baseline volume and the VDT of this nodule (Figure 1), we believe that the diagnostic delay of 6 months would not have been clinically significant because the expected size of the nodule at 12 months (approximately 12 mm) would have led to only a minimal variation in T category (from T1a to T1b).

In a literature search of the PubMed database, we found only one other study published in 2018 in which the performance of the new Fleischner Society guidelines was assessed in a clinical setting [20]. In that study, Scholtz et al. [20] retrospectively evaluated the performance of the new Fleischner Society guidelines for the management of pulmonary nodules incidentally detected during emergent coronary CT angiography. Similar to our study, they found that the application of the new guidelines would have significantly reduced the number of recommended follow-up CT examinations [20]. The main aspect that differentiates the present study from that performed by Scholtz et al. is the sample size (which was smaller in our study); however, in their study, both solid and subsolid (single and multiple) nodules of any size were included. In contrast, only solitary SIPNs with a diameter ≥ 3 mm and volume ≤ 250 mm^3^ were selected in our study. Another aspect that differentiates the two studies is that, in our sample, the CT images and nodule characteristics (type, size, morphology, and location) of all cases were reviewed by an experienced thoracic radiologist, and volumetric analysis using three-dimensional semiautomatic software was performed. In contrast, in Scholtz's study [20], the two-dimensional nodule data were extracted from CT reports, and only in incomplete cases the CT images were reviewed by a board-certified radiologist.

According to the literature [9, 10, 15–19, 21], we found that the patient risk status, nodule margins, and nodule size (for the high-risk group only) were significantly related to the nodule outcome. In contrast to many studies [5, 6, 8–10, 15, 16, 18, 19], no significant relationships were found between the nodule outcome and nodule location; however, we found that 80% of malignant nodules were located in the upper lobes (Table 4).

This study has some limitations. First, it was retrospectively performed. Second, only a small number of SIPNs were included; however, the inclusion criteria were very strict. Third, the image analysis was performed by one observer; however, his experience may have improved the accuracy of the analysis.

## 5. Conclusion

This retrospective study noted that, in the selected sample, the application of the updated Fleischner Society guidelines was effective for managing solitary SIPNs ≤ 250 mm^3^, with a significant reduction in the number of follow-up CT examinations performed, resulting in a significant reduction in the overall radiation dose per patient. We also found that the patient risk status, nodule margins, and nodule size (for the high-risk group only) were significantly related to the nodule outcome.

## Figures and Tables

**Table 1:** Characteristics of the study patients (n = 27).

Characteristics	
Age (years)	65 ± 10.6

Sex	
Male	15 (55.6)
Female	12 (44.4)

Risk status	
Low risk	16 (59.3)
High risk	11 (40.7)

Data are presented as numbers (%) or means ± standard deviations.

**Table 2:** 2017 Fleischner Society management protocol for single solid indeterminate pulmonary nodules ≤ 250 mm^3^.

Nodule volume	Patient risk status	
	Low risk	High risk
< 100 mm^3^	No routine follow-up	Optional CT at 12 months*∗*
100-250 mm^3^	Initial CT at 6-12 month, then consider CT at 18-24 months	Initial CT at 6-12 month, then CT at 18-24 months

*∗* High-risk patients with nodules < 100 mm^3^ with a suspicious morphology and/or an upper lobe location should repeat CT at 12 months.

**Table 3:** Characteristics of the 27 SIPNs ≤ 250 mm^3^ on baseline MDCT in the low- and high-risk groups.

Characteristic	Patient risk status		Chi-square test
	Low risk	High risk	*p *value
Volume			
< 100 mm^3^	8 (29.6)	8 (29.6)	0.247
100-250 mm^3^	8 (29.6)	3 (11.1)	

Margins			
Smooth	13 (48.1)	8 (29.6)	0.383*∗*
Lobulated	3 (11.1)	2 (7.4)	
Spiculated		1 (3.7)	

Lobe location			
Middle/Lower	9 (33.3)	5 (18.5)	0.588
Upper	7 (25.9)	6 (22.2)	

Data are presented as numbers (%).

*∗*Chi-squared test for trend.

**Table 4:** Characteristics and histological features of the malignant nodules.

Nodule	Volume*∗*	Margins*∗*	Lobe location	Time interval 1st-last CT	Volume increment	VDT	Histology	Stage^§^
	(mm^3^)			(days)	(%)	(days)		
1	135	Spiculated	Upper	316	71	407	ADC	IA2
2	55	Smooth	Upper	184	292	93	ADC	IA1
3	80	Lobulated	Upper	411	254	226	SCC	IA2
4	107	Lobulated	Lower	360	75	447	SCC	IA1
5	102	Smooth	Upper	165	175	113	SCC	IA1

*∗*At the baseline MDCT. ^§^According to the 8th edition of the TNM staging.

VDT, volume doubling time; ADC, adenocarcinoma; SCC, squamous cell carcinoma.

**Table 5:** Association between nodule outcome and independent variables (sex, risk status, volume, margins, and lobe location).

Independent variables	Nodule Outcome		Chi-square test
	Stable ≥ 2 years	Malignant	*p *value
Sex			
Male	13 (48.1)	2 (7.4)	0.447
Female	9 (33.3)	3 (11.1)	

Risk status			
Low risk	16 (59.3)	-	0.003
High risk	6 (22.2)	5 (18.5)	

Volume			
< 100 mm^3^	14 (51.9)	2 (7.4)	0.340
100-250 mm^3^	8 (29.6)	3 (11.1)	

Margins			
Smooth	19 (70.4)	2 (7.4)	0.009*∗*
Lobulated	3 (11.1)	2 (7.4)	
Spiculated	-	1 (3.7)	

Lobe location			
Middle/Lower	13 (48.1)	1 (3.7)	0.121
Upper	9 (33.3)	4 (14.8)	

Data are presented as numbers (%).

*∗*Chi-squared test for trend.

## Data Availability

The data used to support the findings of this study are available from the corresponding author upon request.
